# Linear growth failure induced by systemic inflammation inhibiting IGF-1/IGFBP axis in rats with asymptomatic colitis

**DOI:** 10.1186/s12876-019-1023-z

**Published:** 2019-06-20

**Authors:** Xiaoyang Sheng, Xueqing Sun, Feng Li, Junli Wang, Jingqiu Ma

**Affiliations:** 1 Department of Children and Adolescents Health Care, Xin Hua Hospital, School of Medicine, Shanghai Jiao Tong University, Shanghai Institute for Pediatric Research, MOE-Shanghai Key Laboratory of Children’s Environmental Health, No.1665, Kongjiang Road, Yangpu District, Shanghai, 200092 China; 2 Department of Biochemistry and Molecular Cell Biology, Shanghai Jiao Tong University School of Medicine, 280 S. South Chongqing Road, Shanghai, 200025 China; 3 Shanghai Institute for Pediatric Research, Xinhua Hospital, School of Medicine, Shanghai Jiao Tong University, Shanghai Key Laboratory of Pediatric Gastroenterology and Nutrition, No.1665, Kongjiang Road, Yangpu District, Shanghai, 200092 China

**Keywords:** Growth retardation, Asymptomatic colitis, Hormone, Cytokine, Rat

## Abstract

**Background:**

Children in poor areas show significant growth retardation that does not improve with an adequate supply of energy and nutrients, which may be related to asymptomatic intestinal infection caused by poor sanitation. Our objective was to explore the mechanism of intestinal inflammation inhibiting growth in the setting of asymptomatic colitis.

**Methods:**

Forty-eight 3-week-old Wistar rats were randomly divided into three groups: the control group, colitis group (with asymptomatic colitis induced by 2.5% trinitrobenzenesulphonic acid) and pair-fed group (daily food intake matched to the pair in the colitis group). The linear growth was assessed, and the plasma levels of hormone and systemic cytokines were detected and compared by independent two-sample t-test or one-way ANOVA among groups.

**Results:**

At d5, the increases in the body length of the control, colitis and pair-fed groups were 1.65 ± 0.34 cm, 1.10 ± 0.30 cm and 1.38 ± 0.26 cm, respectively, and the increase in the body length in the colitis group was significantly less than that in the control group (*P* < 0.05). There were significant differences in the levels of hormone and cytokines among three groups (*P* < 0.05). Compared with the control group, rats in the colitis group exhibited linear growth failure, as well as higher expression of calprotectin, tumour necrosis factor-α, interleukin-6 and insulin-like growth factor binding protein 2, lower insulin-like growth factor-1 and insulin-like growth factor binding protein 3, and lower expression of nuclear factor kappa B in hepatocytes.

**Conclusions:**

In addition to undernutrition, the systemic inflammatory response caused by asymptomatic colitis may inhibit the linear growth of rats by its influence on the insulin-like growth factor/insulin-like growth factor binding protein axis.

**Electronic supplementary material:**

The online version of this article (10.1186/s12876-019-1023-z) contains supplementary material, which is available to authorized users.

## Background

It is widely recognized that infants and young children in impoverished areas of developing countries have persistently high rates of growth stunting, and undernutrition has long been considered the most important cause. However, recently, it has been increasingly realized that, although an adequate supply of energy and nutrients is clearly necessary, it is not sufficient to ensure normal linear growth [1]. Furthermore, some recent studies performed in poor areas have indicated the combined usage of zinc and multiple micronutrients did not improve growth faltering in infants and children [2, 3]. Children aged less than 2 years, whose growth is rapid, are particularly vulnerable to environmental factors. Infants and young children living in conditions of poor sanitation are frequently exposed to pathogenic microbes, resulting in a substantial underlying burden of chronic and asymptomatic intestinal infection [4, 5].

The systemic inflammatory process induced by intestinal infection may suppress the growth hormone (GH)/insulin-like growth factor-1 (IGF-1)/IGF binding protein (IGFBP) axis, which is essential for normal linear growth, and may also have an impact on the effect of complementary feeding interventions in infants and young children [6]. However, the role of pro-inflammatory cytokines abnormally elevated during inflammation in disturbing the GH/IGF-1/IGFBP axis remains inconsistent [7–10]. Furthermore, a previous study of our research group carried out in Xichou County, Yunnan Province, China, a rural community, found that the length-for-age Z score (LAZ) of 6-month-old infants was substantially negative and that 1 year of nutritional intervention did not improve the growth faltering of rural infants [11]. Notably, the concentrations of faecal calprotectin, an established noninvasive biomarker of intestinal inflammation [12, 13], were increased significantly in rural healthy infants and were negatively correlated with the LAZ [14]. It was suggested that calprotectin may play a role in growth faltering. However, thus far, no study has explored the role of calprotectin in inhibiting growth and its mechanism.

A previous study concluded that inflammation itself has a harmful effect on linear growth, likely due, in part, to a reduction in the plasma concentrations of IGF-1 through a rat colitis model induced by trinitrobenzenesulphonic acid (TNBS) [15]. Nevertheless, the establishment of a colitis model was based on inflammatory bowel disease (IBD), a group of intestinal diseases with obvious clinical symptoms such as diarrhoea, pain and weight loss. Another murine intestinal infection model induced by *Citrobacter rodentium* suggested that linear growth failure was associated with systemic inflammation and suppressed serum levels of IGF-1. However, all mice with infectious colitis exhibited obvious diarrhoea during the experiment [16].

In our study, we established a rat model through TNBS enema in which rats had asymptomatic colitis similar to the potential intestinal infection caused by poor sanitation in children living in poverty areas. Using the rat model of asymptomatic colitis, the aims of our study were to explore 1) linear growth failure of rats, 2) changes in the faecal calprotectin and plasma levels of hormone and systemic cytokines, and 3) the relationship among linear growth, plasma hormone and cytokines.

## Methods

### Animals

Forty-eight 3-week-old male specific-pathogen-free Wistar rats (approximately 75 g to 95 g in body weight) were used in this study and were purchased from Shanghai SLAC Laboratory Animal Co., Ltd. (Shanghai, China). The rats were housed individually at an ambient temperature of (22 ± 1)°C, were maintained under a 12-h light-dark cycle and were given standard laboratory chow and tap water. All the rats were anaesthetized by intraperitoneal injection of 1% pentobarbital sodium (0.01 mL/g) (Sigma Chemical Co., UK) before each operation to minimize animal suffering.

### Colitis model

The rats were randomly divided into three groups (*n* = 16/group): 1) free-feeding sham-operation control group (abbreviated as the control group), 2) 2,4,6-trinitrobenzenesulphonic acid (TNBS) colitis group (abbreviated as the colitis group) and 3) pair-fed sham-operation group (abbreviated as the pair-fed group). Rats in the control group were allowed free access to food, while the daily food intake of the pair-fed group matched that of their pairs in the colitis group. To achieve the precise matching of food intake, the pair-fed group was started 1 day after the colitis group [15]. Colitis was induced by the children’s stomach tube inserted 5 cm proximal to the anus together with a little glycerol as lubricant. After the rats were anaesthetized by pentobarbital sodium (1%, 0.01 mL/g), a volume of 0.1 mL of a solution of 2.5% TNBS (5% TNBS; Sigma Chemical Co. UK; half diluted in Phosphate Buffered Saline (PBS)) was injected into the colon, and this step was repeated every 2 days in the colitis group. In the control and pair-fed groups, a volume of 0.1 mL of PBS (0.01 M, pH 7.4) was injected into the colon as the sham operation. The rats were measured for their weight and length on the operation day (marked as d0), 3rd day (marked as d3) and 5th day (marked as d5). The rats’ body length, assessed by the mean of two measurements of the nose-to-tail base distance, was measured after being anaesthetized before sacrifice [15]. The previous 24 h of food intake was measured daily in all rats.

At d3 and d5, 24 rats (8 for each group) were anaesthetized by a dose of pentobarbital sodium (1%, 0.015 mL/g). The rats were fixed in the supine position, and approximately 1.5 mL of blood from the heart was collected into EDTA tubes. Next, each rat was perfused transcardially with 150 mL of PBS, followed by 200 mL of 4% paraformaldehyde in 0.01 M phosphate buffer (pH 7.4). During this perfusion process, the rats were mercifully killed without any pain. The blood was centrifuged at 4 °C at a speed of 3000 rpm for 15 min, and the plasma was stored at − 80 °C until ELISA. Using midline laparotomy, approximately 0.5 cm^3^ of the liver tissue was fixed in 4% ice-cold paraformaldehyde for 24 h for immunohistochemistry. The 7-cm colon proximal to the anus was removed and opened longitudinally, and faeces in the colon section were stored at − 20 °C for calprotectin detection. After the colon section was rinsed with PBS and exposed, macroscopic inflammation was assessed, and then the colon was cut longitudinally into two halves, one half was weighed and five times the volume of RIPA (Thermo) was added for homogenization. The colon homogenate was first centrifuged for 20 min at 2100 rpm, and then the supernatant was recentrifuged for 15 min at 12000 rpm and stored at − 80 °C until assayed for myeloperoxidase (MPO) concentrations. The remaining half of the colon was fixed in 4% ice-cold paraformaldehyde for 24 h for histopathologic staining. Our animal experiments complied with the ARRIVE guidelines.

### Macroscopic score

Macroscopic damage of the colonic mucosa was assessed by two independent observers who were unaware of the animals’ treatment. The scale for macroscopic damage ranged from 0 (normal) to 5 (severe) and was based on hyperaemia, sites of ulceration, and sites of inflammation according to the criteria of Morris (Additional file 1**:** Table S1) [17].

### Pathology and immunohistochemistry

The rats’ colon samples were fixed in 4% paraformaldehyde, embedded in paraffin, sectioned and stained with haematoxylin and eosin (HE).

Immunohistochemical staining of paraffin-embedded liver tissues was performed by incubating with the primary antibodies anti-rat growth hormone receptor (GHR) (#119–12,770; 1:100 dilution; RayBiotech) and nuclear factor kappa B (NF-κB) p65 (#ab13594; 1:100 dilution; Abcam) overnight at 4 °C. After the incubation with the biotin-conjugated 2nd antibody for 2 hours at room temperature, the samples were treated with ABC Elite immunoperoxidase (Vector Laboratories) and DAB substrate (Vector Laboratories) according to the manufacturers’ instructions and were finally stained with haematoxylin. The analysis and semi-quantification of positive immunostaining were performed using Image-Pro Plus v7.0 software (Media Cybernetics Inc., USA) using the parameter of the mean density. All images were captured using a CoolSNAP colour camera (Media Cybernetics Inc., USA) at a magnification of 200× in an Olympus BX51 microscope.

### Myeloperoxidase (MPO) concentrations

The tissue concentrations of MPO were measured to assess the degree of intestinal inflammation using the MPO colorimetric activity assay kit (#MAK068; Sigma-Aldrich, St. Louis, USA). Fifty microliters of 1:40-diluted colon homogeneity samples was added to 50 microliters of MPO substrate according to the manufacturer’s instructions. The reaction was stopped after 60 min at room temperature away from light, and the absorbance at 412 nm was measured by the microplate reader (Molecular Device, USA). The amount of TNB consumed by the enzyme assay were determined by the difference value between each sample blank and its corresponding samples (ΔA_412_ = (A412)_sample blank_-(A412)_sample_, then we should compare theΔA_412_ of each sample to the standard curve to get the amount of TNB consumed. The MPO activity was calculated by the following equation:

$$ MPO\  activity=\frac{B\times Sample\ Dilution\ Factor}{\left(\mathrm{Reaction}\ \mathrm{Time}\right)\times \mathrm{V}} $$

where B = amount of chromophore TNB consumed, and V = sample volume added to well. All the samples were analysed in 3 wells in each experiment, and each experiment was repeated 3 times.

## Elisa

The concentrations of plasma and faecal calprotectin (#K6936; Immundiagnostik, Bensheim, Germany), plasma concentrations of IL-6 (#EZRIL6; Millipore, Boston, MA, USA), TNF-α (#ELR-TNFa; RayBiotech, Norcross, GA, USA), IGF-1 (#ELR-IGF1; RayBiotech, Norcross, GA, USA), IGF-binding protein-3 (IGFBP3) (#80581; Crystal Chem, Downers Grove, USA), IGFBP2 (#ab207615; Abcam, Cambridge, MA, USA) and GH (#EZRMGH-45 K; Millipore, Boston, MA, USA) were determined by ELISA. All the experiments were performed according to the manufacturer’s instructions.

### Statistical analysis

SPSS 13.0 statistical software was used for all descriptive analyses. The results were presented as the means ± SD for normally distributed continuous variables or medians (interquartile range) for data not normally distributed. Linear growth was presented as the change in the body length during the five-day experimental period. Independent two-sample t-test and one-way ANOVA were used to compare normally distributed continuous variables among the experimental groups, and skewed data were compared after log transformations by ANOVA. The LSD multiple comparison test was used for the post hoc test if the overall ANOVA had a *P* value less than 0.05. Chi-square test was used for categorical variables. Pearson’s correlation was used to test for a linear relationship between two quantitative variables (skewed data were analysed after log transformation). All statistical tests were two-tailed, and *P* values < 0.05 were considered statistically significant.

## Results

### General conditions

The rats in the three groups appeared well, with a flexible response, glossy hair, active foraging and no significant reduction in activity. They all had solid stools and no diarrhoea throughout the course of experiment. There was no death in all rats.

### Macroscopic and pathological findings

The macroscopic findings showed hyperaemia and oedema in the colonic mucosa in the rats of both the colitis and pair-fed groups, suggesting a low level of colonic damage (score ≤ 1). There was no detectable macroscopic injury in the colons of rats from the control group.

**Fig. 1:**
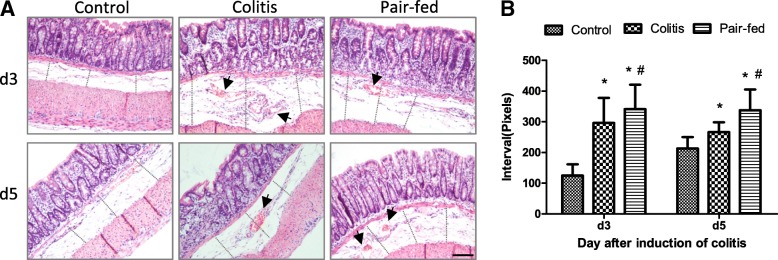
Pathological findings of the colonic mucosa of rats in the three groups. At d3 and d5, the colonic villus was intact by HE staining in all three groups. Hemangiectasis was found in both the colitis and pair-fed groups (**a**); more vessels were found and indicated by arrows. Furthermore, in both the colitis and pair-fed groups, the basal layer under the epithelium was obviously loose and the space between the muscle and epithelium layers was larger than that of the control group (**a** and **b**). Each figure was measured for three times at intervals (the space between the muscle and epithelium layers) as indicated by dotted line in (**a**) and all data of intervals were analyzed in (**b**). Scale bar = 100 μm. * *P* < 0.05 vs control group; # *P* < 0.05 vs colitis group

The pathological findings showed that the colonic villus was intact after HE staining in all three groups. In both the colitis and pair-fed groups, the basal layer under the epithelium was obviously loose and the space between the muscle and epithelium layer was larger than that in the control group (Fig. 1a, b). Hemangiectasis was found in both the colitis and pair-fed groups at d3 and d5 (Fig. 1a).

### Myeloperoxidase (MPO) in colonic tissue

**Fig. 2:**
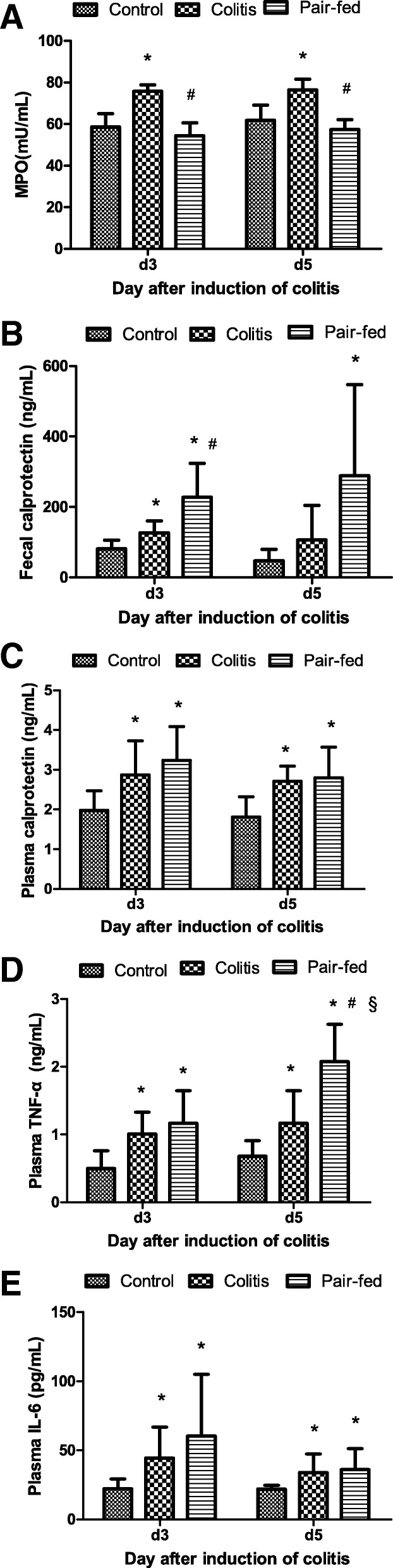
Comparison of MPO in colonic tissue and cytokine levels among the three groups at d3 and d5. The values are expressed as the means (SD), *n* = 8/group. **a** Intestinal MPO concentration. **b** Faecal calprotectin levels. **c** Plasma calprotectin concentrations. **d** Plasma TNF-α concentrations. **e** Plasma IL-6 concentrations. The skewed data of faecal calprotectin, TNF-α and IL-6 were compared after log transformations among the three groups by ANOVA. * *P* < 0.05 vs control group; # *P* < 0.05 vs colitis group; §*P* < 0.05 vs d3

The MPO activity is an indicator of the infiltration of the colon with polymorphonuclear leukocytes caused by the TNBS. There were significant differences in the colonic MPO concentrations among the three groups at d3 and d5 (*F* = 34.49, *P* < 0.001; *F* = 23.67, *P* < 0.001), and the colonic MPO concentrations in the colitis group were higher than those in the control group or pair-fed group (*P* < 0.01) (Fig. 2). However, the difference in the MPO concentrations between the control and pair-fed groups was not significant (*P* > 0.05) (Fig. 2).

### Comparison of the cytokine levels among the three groups

At d3 and d5, the faecal calprotectin concentrations among the three groups were significantly different (*F* = 19.09, *P* < 0.001; *F* = 4.35, *P* = 0.026). At d3, the faecal calprotectin levels were increased in both the colitis and pair-fed groups compared with those in the control group (*P* < 0.05), and the rats in the pair-fed group had significantly higher faecal calprotectin levels than those in the colitis group (*P* < 0.05). At d5, the faecal calprotectin levels in the pair-fed group were significantly higher than those in the control group (*P* < 0.05) (Fig. 2).

At d3 and d5, the plasma calprotectin concentrations among three groups were significantly different (*F* = 5.92, *P* = 0.009; *F* = 7.16, *P* = 0.004) and were significantly higher in the colitis and pair-fed groups than in the control group (*P* < 0.05) (Fig. 2).

At d3 and d5, there were significant differences in the plasma TNF-α concentrations among the three groups (*F =* 4.44, *P =* 0.025*; F =* 20.02, *P <* 0.001) that were significantly higher in the colitis and pair-fed groups than in the control group (*P* < 0.05) at d3 (Fig. 2). At d5, the TNF-α concentrations were increased in the pair-fed group compared with those at d3 (*t* = 2.79, *P* = 0.014) and were significantly higher than those in the colitis and control groups (*P* < 0.05). Furthermore, the rats in the colitis group had significantly higher TNF-α concentrations than those in the control group (*P* < 0.05) at d5 (Fig. 2).

At d3 and d5, significant differences were found in the plasma IL-6 concentrations among the three groups (*F =* 6.07, *P =* 0.008*; F =* 3.90, *P =* 0.036), and the rats in the colitis and pair-fed groups had significantly higher IL-6 concentrations than those in the control group (*P* < 0.05) (Fig. 2).

### Food intake

The daily food intake of rats in the pair-fed group was matched to the pair in the colitis group; thus, the food intake was only compared between the control and colitis groups.

**Fig. 3:**
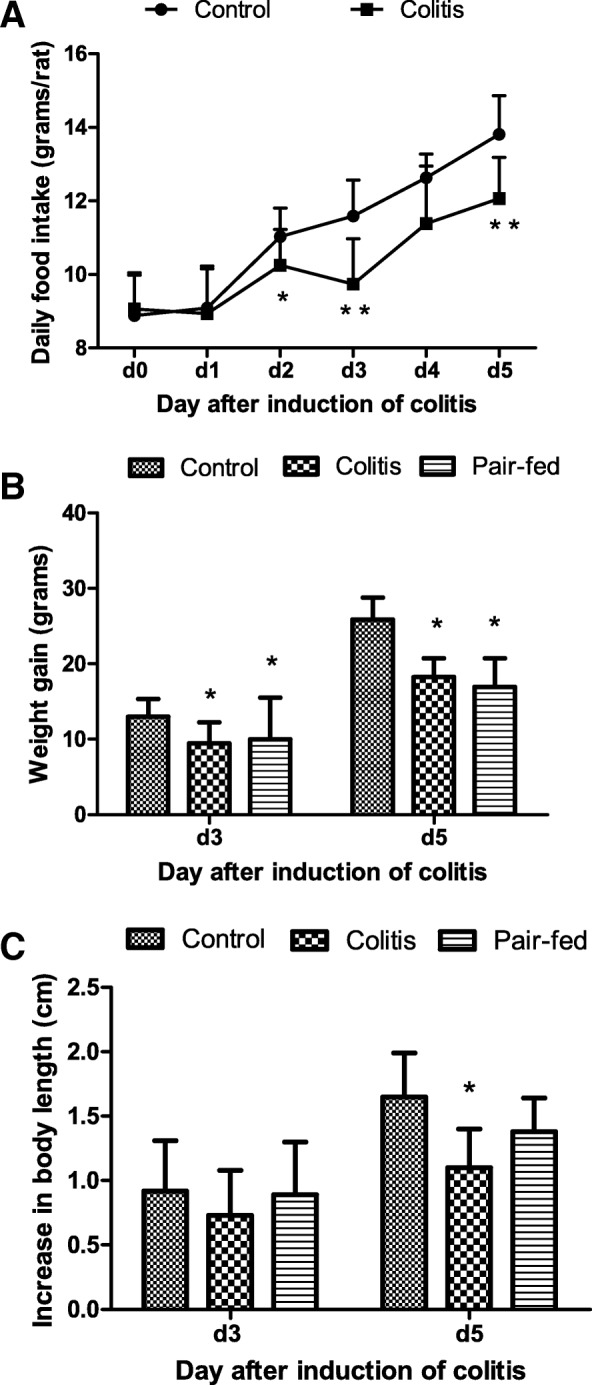
Comparison of the daily food intake, weight gain and increase in the body length among the three groups. The values are expressed as the means (SD). **a** Daily food intake. **b** Weight gain. **c** Increase in body length. * *P* < 0.05 vs control group; ** *P* < 0.01 vs control group. By design, food intake in the pair-fed group was the same as that in the colitis group

The food intake of rats in the control group was increased gradually over time. However, the food intake in the colitis group was increased slowly after enema and was decreased obviously at d3. The rats in the colitis group ate significantly less than those in the control group at d2, d3 and d5 (*t* = 2.51, *P =* 0.018; *t* = 4.74, *P* < 0.001; *t* = 3.22, *P =* 0.006) (Fig. 3).

### Weight gain and linear growth of rats

At d3 and d5, the weight gain (weight at d3 or d5 subtracts weight at d0) was significantly different among the three groups (*F* = 3.94, *P* = 0.026; *F* = 19.22, *P* < 0.001) and the weight gain in the colitis group and pair-fed group was significantly less than that in the control group (*P* < 0.05) **(**Fig. 3**)**.

At d5, the increases in the body length of the control, colitis and pair-fed groups were 1.65 ± 0.34 cm, 1.10 ± 0.30 cm and 1.38 ± 0.26 cm, respectively, and there was a significant difference among the three groups (*F* = 6.55, *P* = 0.006). The increase in the body length of rats in the colitis group was significantly less than that in the control group (*P* < 0.05) (Fig. 3).

### Plasma hormone concentrations

**Fig. 4:**
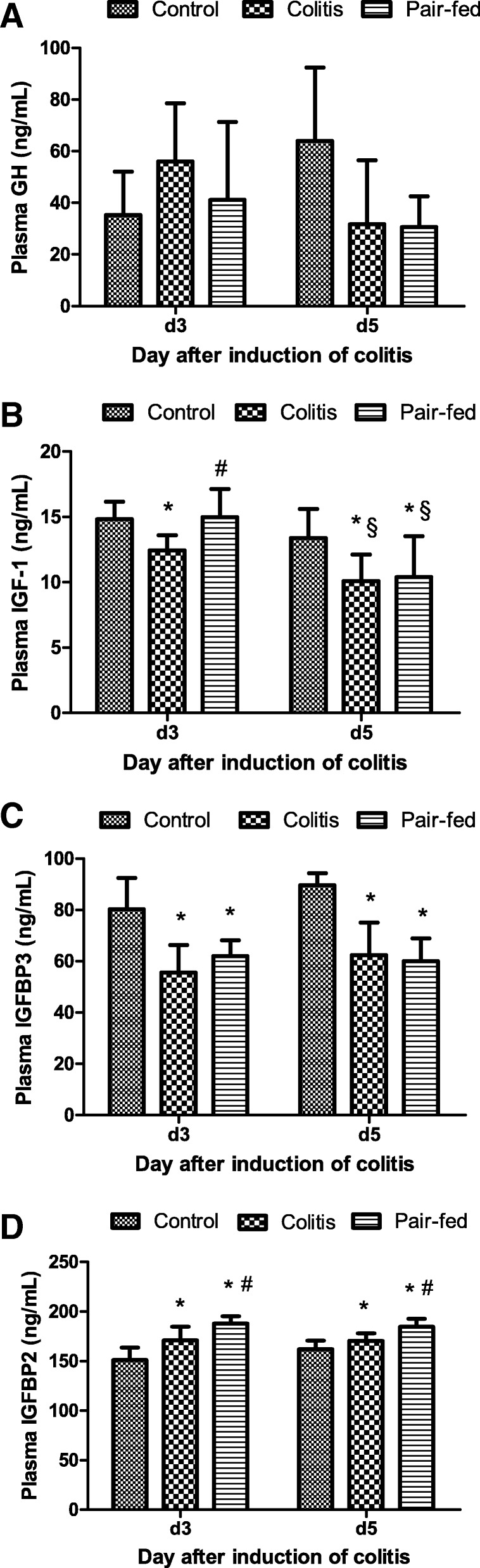
Comparison of the plasma hormone concentrations among the three groups at d3 and d5. The values are expressed as the means (SD), *n* = 8/group. **a** Plasma GH. **b** Plasma IGF-1. **c** Plasma IGFBP3. **d** Plasma IGFBP2. Skewed data of plasma GH are compared after log transformations among three groups by ANOVA. *P < 0.05 vs control group; # *P* < 0.05 vs colitis group; §*P* < 0.05 vs d3

There was no significant difference in the plasma GH concentrations among the three groups at d3 and d5 (*P* = 0.173; *P* = 0.082) (Fig. 4).

At d3 and d5, there were significant differences in the plasma IGF-1 concentrations among the three groups (*F* = 6.12, *P =* 0.008; *F* = 4.29, *P =* 0.027). At d3, the IGF-1 concentrations were significantly lower in the colitis group than those in the control group and pair-fed group (*P* < 0.05) (Fig. 4). At d5, the IGF-1 concentrations in both the colitis and pair-fed groups were further decreased compared with those at d3 (*t* = 2.89, *P* = 0.012; *t* = 3.42, *P* = 0.004) and were significantly lower than those in the control group (*P* < 0.05) (Fig. 4).

The plasma IGFBP3 concentrations were significantly different among the three groups at d3 and d5 (*F* = 13.22, *P* < 0.001; *F* = 24.76, *P* < 0.001) and were significantly lower in both the colitis and pair-fed groups than those in the control group (*P* < 0.05) (Fig. 4). At d3 and d5, the plasma IGFBP2 concentrations were significantly different among the three groups (*F* = 19.90, *P* < 0.001; *F* = 15.80, *P* < 0.001) and were significantly higher in both the colitis and pair-fed groups than those in the control group (*P* < 0.05). Additionally, the rats in the pair-fed group had significantly higher IGFBP2 concentrations than those in the colitis group (*P* < 0.05) (Fig. 4).

### Relationship among linear growth, plasma hormone and cytokines

The relationship among linear growth, plasma hormone and cytokines of all 48 rats were analysed. The results of Pearson’s correlation indicated that both the faecal and plasma concentrations of calprotectin were negatively correlated with the increase in the body length at d5 (respectively: *r* = − 0.501, *P* = 0.013; *r* = − 0.469, *P* = 0.021). Furthermore, at d5, the TNF-α concentrations were negatively correlated with the IGF-1 concentrations (*r* = − 0.424, *P* = 0.039). At d5, the IGFBP2 concentrations were positively correlated with the plasma concentrations of IL-6, TNF-α and calprotectin (respectively: *r* = 0.461, *P* = 0.023; *r* = 0.629, *P* = 0.001; *r* = 0.488, *P* = 0.016), while the IGFBP3 concentrations were negatively correlated with the plasma concentrations of IL-6, TNF-α and calprotectin and faecal concentrations of calprotectin (respectively: *r* = − 0.630, *P* = 0.001; *r* = − 0.801, *P <* 0.001; *r* = − 0.711, *P <* 0.001; *r* = − 0.593, *P* = 0.002).

The IGF-1 concentrations were positively correlated with the increase in the body length at d5 (*r* = 0.419, *P* = 0.042). At d3, it was also found that the calprotectin concentrations in faeces were positively correlated with those in plasma (*r* = 0.532, *P* = 0.007).

### Expression of NF-κB and GHR proteins in hepatocytes, as determined by immunohistochemistry

**Fig. 5:**
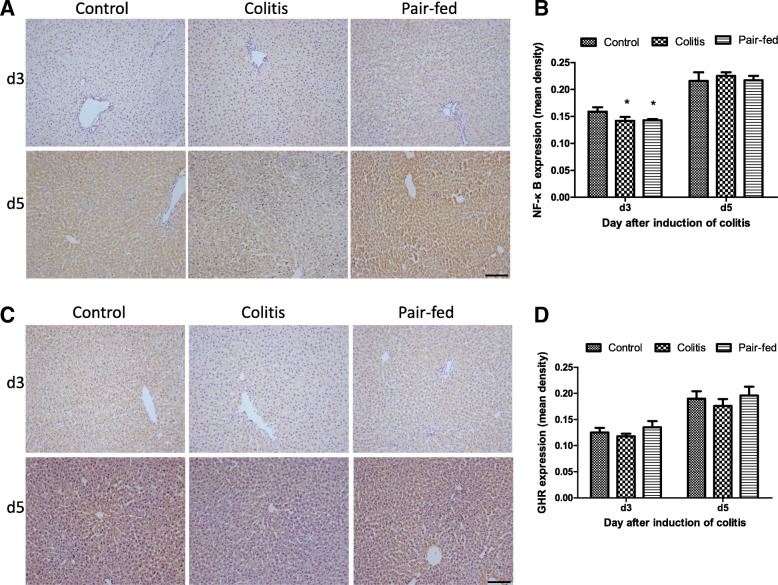
Expression of GHR and NF-κB proteins in hepatocytes as determined by immunohistochemistry. **a** and **c** Representative images of the immunohistochemical analysis of GHR and NF-κB proteins in hepatocytes. **b** and **d** The expression levels of GHR and NF-κB proteins (mean density) in hepatocytes were compared among the three groups at d3 and d5. Scale bar = 100 μm. * *P* < 0.05 vs control group

The expression of NF-κB proteins (mean density) in hepatocytes was significantly different among the three groups at d3 (*F* = 9.31, *P* = 0.006) and was significantly lower in both the colitis and pair-fed groups than in the control group (*P* < 0.05). Furthermore, NF-κB protein expression was similar between the colitis and pair-fed groups (*P* > 0.05). At d5, NF-κB protein expression was increased in both the colitis and pair-fed groups, and no significant difference was found in the NF-κB protein expression among the three groups (*P* > 0.05). No significant differences were found in the expression of GHR proteins in hepatocytes among the three groups (*P* > 0.05) at d3 and d5 (Fig. 5).

At d3, NF-κB protein expression was negatively correlated with the plasma concentrations of TNF-α and faecal concentrations of calprotectin (respectively: *r* = − 0.678, *P* = 0.015; *r* = − 0.631, *P* = 0.028).

## Discussion

In order to mimic asymptomatic intestinal inflammation in children in poor areas caused by poor sanitation, based on the results of preliminary experiments, in this study, we established the asymptomatic colitis model using a 2.5% low enema concentration of TNBS, approximately half of the TNBS concentrations used in other colitis models [15, 18]. Furthermore, in order to reduce the severity of intestinal inflammation, we did not use ethanol as the “barrier breaker”, but increased the frequency of TNBS enema (every 2 days). Thus, no ulcer and only hyperaemia and oedema were found in the colonic mucosa of the colitis group by macroscopic observation. Pathological findings also indicated only mild damage in the colonic mucosa. The colonic MPO concentrations in the colitis group were significantly increased. Moreover, we found an obvious decrease in the body length increase and less weight gain 5 days after TNBS enema compared with that in the control group. Therefore, this model may serve as a novel means of assessing the mechanisms of linear growth suppression during asymptomatic colitis.

In the model, the sensitive biomarker of intestinal inflammation, faecal calprotectin, was significantly increased after TNBS administration, indicating a certain degree of colonic inflammation occurred in the colitis model. Furthermore, the plasma levels of calprotectin, TNF-α and IL-6 were also significantly increased, suggesting a systemic inflammatory response in the rats. An increased plasma concentration of calprotectin (also called S100A8/A9) correlates well with disease activity in many inflammatory conditions and is already accepted as a superior biomarker to other inflammation markers such as C-reactive protein (CRP) [19, 20]. Furthermore, calprotectin is not only a useful marker of inflammation but also plays a pivotal role in the pathogenesis of inflammatory disorders [21, 22]. The concentration of calprotectin in faeces is approximately 6 times that in plasma in healthy adults [23]. In this study, a positive correlation was also found between the calprotectin concentrations in faeces and plasma. Faecal calprotectin has been widely used in the detection of intestinal inflammation. Due to the simplicity of collection, storage and detection, compared with plasma calprotectin, faecal calprotectin has the special advantage in infants and young children of not requiring venepuncture. In a previous study, we found a significantly negative correlation between faecal calprotectin and linear growth in rural infants [14]. In this study, we confirmed this relationship again by establishing animal models. It was suggested that the elevated calprotectin may have a direct inhibitory effect on the linear growth of children. Therefore, it is necessary to further explore the mechanism of calprotectin inhibiting growth, in the hope that faecal calprotectin will be used as an important screening indicator in the future to identify intestinal inflammation-induced growth retardation for sub-healthy children in poor rural areas.

Normal growth is largely dependent on the GH-IGF-IGFBP axis, and any factor impairing the GH-IGF-IGFBP axis will produce adverse effects on linear growth. To explore the relationship between systemic inflammation and linear growth, the plasma levels of GH, IGF-1, IGFBP2 and IGFBP3 were detected, and further expression of NF-κB and GHR proteins in hepatocytes were also determined. Thus, we found that the IGF-1 level in rats in the colitis group decreased significantly at d3 and further decreased at d5. Moreover, we observed a transient decrease in NF-κB protein expression in hepatocytes in the colitis group that was negatively correlated with the faecal calprotectin concentrations at d3. NF-κB is a critical signalling molecule in the inflammatory process that not only exerts a pro-inflammatory effect but also plays an anti-inflammatory role [24] and may mediate the inhibitory effect of TNF-α on IGF [25, 26]. Our results suggested that calprotectin might have an inhibitory effect on IGF-1 production via the NF-κB-mediated signalling pathway.

Additionally, both decreased IGFBP3 and increased IGFBP2 levels were found in the colitis group, and the levels of two binding proteins were significantly correlated with calprotectin. Most of the circulating IGF-1 protein is in complex with acid labile subunit (ALS) and IGFBP-3, which increases its half-life by 100 fold. Elevated inflammatory cytokines may increase the proteolysis of IGFBP-3 and impair the formation of the IGF-1/IGFBP-3/ALS complex, resulting in a shorter IGF-1 half-life and enhanced clearance of IGF-1 [7]. Furthermore, increased levels of IGFBP-2 have previously been described in paediatric IBD patients [27]. The mitogenic effect of IGF-1 was strongly reduced by the exogenous administration of IGFBP-2 in human growth plate chondrocytes [28]. Thus, the reduction in the IGF-1 concentration may be partly attributed to the influence of abnormally elevated calprotectin on IGFBP3 and IGFBP2. However, a significant correlation between two variables does not indicate a cause-and-effect relationship. In future research we will use antibody blockade method to further elucidate underlying mechanisms of calprotectin inhibiting linear growth.

In our study, elevated IL-6 and TNF-α may also inhibit the linear growth of rats by the influence on IGFBP2 and IGFBP3. Moreover, the negative correlation between TNF-α and IGF-1 has indicated TNF-α might have a direct inhibitory effect on IGF-1, similar to that observed in other animal studies [29, 30].

Animal studies have shown that inflammatory cytokines could induce hepatic GH resistance [7, 8]. However, as opposed to GH resistance, GH secretion was also reported to be normal or low in children with inflammatory growth retardation in IBD [31]. In our study, there was no GH resistance associated with elevated inflammatory cytokines.

Additionally, the colonic mucosa of the rats in the pair-fed group also showed a similar and pathological manifestation to that of the colitis group that may be attributed to the inadequate intake of proteins and carbohydrates. However, TNF-α and faecal calprotectin in the pair-fed group were increased as high as or even higher than those in the colitis group. Thus, we further evaluated the expression of autophagy marker protein light chain 3 (LC3) in hepatocytes [32] by immunohistochemistry and found it was increased in the pair-fed group compared with that in other two groups, although no significant difference was found among the three groups (*P* = 0.081, data not shown). In view of this, the involvement of starvation-induced autophagy should be considered [33].

## Conclusions

**Fig. 6:**
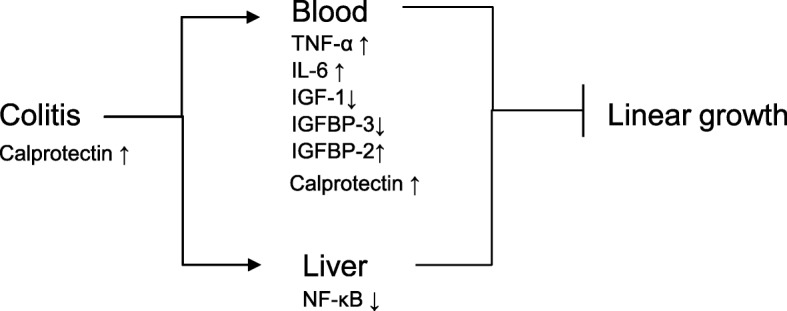
Systemic inflammatory response induced by asymptomatic colitis may partly cause linear growth retardation by the influence on the IGF/IGFBP axis

This study was the first to establish an asymptomatic colitis model mimicking the growth pattern observed in early childhood in poor areas where the children are at high risk of intestinal inflammation. Furthermore, this study is the first to explore the relationship among linear growth, plasma hormone and calprotectin. In addition to the undernutrition, the systemic inflammatory response caused by asymptomatic colitis may also aggravate the linear growth retardation by the influence on the IGF/IGFBP axis **(**Fig. 6**)**.

## Additional file

Additional file 1: **Table S1.** Criteria for assessment of colonic damage induced by TNBS. (DOCX 16 kb)

## Data Availability

All data generated or analysed during this study are included in this published article.

## Abbreviations

CRP: C-reactive protein
GH: Growth hormone
GHR: Growth hormone receptor
IBD: Inflammatory bowel disease
IGF-1: Insulin-like growth factor-1
IGFBP2: Insulin-like growth factor binding protein 2
IGFBP3: Insulin-like growth factor binding protein 3
IL-6: Interleukin-6
LAZ: Length-for-age Z score
MPO: Myeloperoxidase
NF-κB: Nuclear factor kappa B
TNBS: Trinitrobenzenesulphonic acid
TNF-α: Tumour necrosis factor-α
