# Supplementary material for: Linear growth failure induced by systemic inflammation inhibiting IGF-1/IGFBP axis in rats with asymptomatic colitis
Source: BMC Gastroenterol. 2019 Jun 20;19:96. doi: 10.1186/s12876-019-1023-z (PMC6585116; doi:10.1186/s12876-019-1023-z)
Supplement: Supplementary file 1 — Table S1. Criteria for assessment of colonic damage induced by TNBS. (DOCX 16 kb) [file 12876_2019_1023_MOESM1_ESM.docx]

**TABLE S1** Criteria for assessment of colonic damage induced by TNBS

| Score | Colonic feature |
| --- | --- |
| 0 | No damage. |
| 1 | One region of localized inflammation or thickening; No ulcers. |
| 2 | Linear ulceration, but no significant inflammation. |
| 3 | Linear ulceration with inflammation at one site. |
| 4 | Two or more sites of ulceration and/or inflammation; ulcers present in at least one site. |
| 5 | Two or more sites of ulceration and inflammation with one major site of ulceration and inflammation extending >1 cm along the length of the colon. |
